# Maternal cadmium, iron and zinc levels, DNA methylation and birth weight

**DOI:** 10.1186/s40360-015-0020-2

**Published:** 2015-07-15

**Authors:** Adriana C. Vidal, Viktoriya Semenova, Thomas Darrah, Avner Vengosh, Zhiqing Huang, Katherine King, Monica D. Nye, Rebecca Fry, David Skaar, Rachel Maguire, Amy Murtha, Joellen Schildkraut, Susan Murphy, Cathrine Hoyo

**Affiliations:** Department of Surgery, Division of Urology, Cedars-Sinai Medical Center, Los Angeles, CA 90048 USA; Department of Biological Sciences, Center for Human Health and the Environment, North Carolina State University, Raleigh, NC 27695 USA; Department of Public Health, Brody School of Medicine, East Carolina University, Greenville, NC 27834 USA; Division of Water, Climate, and the Environment, School of Earth Sciences, The Ohio State University, Columbus, OH 43210 USA; Nicholas School of the Environment, Duke University, Research Drive, Durham, NC 27710 USA; Division of Gynecologic Oncology, Department of Obstetrics and Gynecology, Duke University School of Medicine, Research Drive, Durham, NC 27710 USA; Environmental Public Health Division, U.S. Environmental Protection Agency, Chapel Hill, NC 27599 USA; University of North Carolina at Chapel Hill, Lineberger Comprehensive Cancer Center, 450 West Drive, Chapel Hill, NC 27599 USA; Department of Environmental Sciences and Engineering, Gillings School of Global Public Health, UNC-Chapel Hill, Chapel Hill, NC 27599 USA; Division of Maternal Fetal Medicine, Department of Obstetrics and Gynecology, Duke University School of Medicine, Erwin Drive, Durham, NC 27710 USA; Department of Community and Family Medicine and Duke Cancer Institute, Duke University School of Medicine, Erwin Drive, Durham, NC 27710 USA

**Keywords:** Cadmium, Zinc, Genomic imprinting, Epigenetics, Pediatrics, Obesity

## Abstract

**Background:**

Cadmium (Cd) is a ubiquitous and environmentally persistent toxic metal that has been implicated in neurotoxicity, carcinogenesis and obesity and essential metals including zinc (Zn) and iron (Fe) may alter these outcomes. However mechanisms underlying these relationships remain limited.

**Methods:**

We examined whether maternal Cd levels during early pregnancy were associated with offspring DNA methylation at regulatory sequences of genomically imprinted genes and weight at birth, and whether Fe and Zn altered these associations. Cd, Fe and Zn were measured in maternal blood of 319 women ≤12 weeks gestation. Offspring umbilical cord blood leukocyte DNA methylation at regulatory differentially methylated regions (DMRs) of 8 imprinted genes was measured using bisulfite pyrosequencing. Regression models were used to examine the relationships among Cd, Fe, Zn, and DMR methylation and birth weight.

**Results:**

Elevated maternal blood Cd levels were associated with lower birth weight (*p* = 0.03). Higher maternal blood Cd levels were also associated with lower offspring methylation at the *PEG3* DMR in females (β = 0.55, se = 0.17, *p* = 0.05), and at the *MEG3* DMR in males (β = 0.72, se = 0.3, *p* = 0.08), however the latter association was not statistically significant. Associations between Cd and *PEG3* and *PLAGL1* DNA methylation were stronger in infants born to women with low concentrations of Fe (*p* < 0.05).

**Conclusions:**

Our data suggest the association between pre-natal Cd and offspring DNA methylation at regulatory sequences of imprinted genes may be sex- and gene-specific. Essential metals such as Zn may mitigate DNA methylation response to Cd exposure. Larger studies are required.

## Background

Cadmium (Cd) is a naturally occurring toxic group IIb transition metal that is ubiquitous in the earth’s crust. Increased anthropogenic utilization of Cd in the last several decades has led to increased human exposure at high doses; exposure vectors are wide ranging, including waste and emissions from mining, smelting, industrial activities, sewage sludge, tobacco smoking and fruit and vegetables contaminated by the use of phosphate fertilizers in agriculture [1]. Cd is a nephrotoxin, neurotoxicant, osteotoxicant, and carcinogen [2]. Cellular effects include apoptosis, DNA fragmentation and chromatin structural changes. Cd exposure has also been implicated in the etiology of fetal growth restriction [1, 3–5].

Zinc (Zn), and iron (Fe) are essential metals found in wheat, seeds, beans, seafood, and red meats as well as dietary supplements, and over-the-counter drugs [6]. Because Zn and Fe are co-factor for numerous enzymes involved in nucleic acid synthesis and repair, they play a significant role in growth, development, and cellular functions [7, 8]. Animal and cross-sectional human data suggest that at moderate levels, Zn and Fe may mitigate Cd effects via trans-metallation processes; however empirical data remain limited in human.

*In vitro* and *in vivo* studies demonstrated that exposure to Cd modifies DNA methylation patterns [9–12], although specific targets remain largely unknown. In humans, epigenetic targets for Cd exposure identified from unbiased approaches lack specificity, and the malleability of these epigenetic marks remains unknown. Moreover, although the timing of exposure during the life course is critical in determining severity of effects following exposure, human data is primarily from cross-sectional studies and reports in adults.

Parental allele-specific differentially methylated regions (DMRs) of imprinted genes are established in the gametes and in the early embryo, and are generally stable in tissues from all germ layers. Perturbations in the establishment or maintenance of these DMRs during early embryogenesis can result in systemic variability that may be detected in nearly any tissue [13, 14]. Aberrant methylation at some imprint regulatory regions has been associated with lower birth weight [15].

In the present analysis, we examine the association between levels of Zn, Fe and Cd in pregnant women and DNA methylation at imprinted regulatory DMRs in umbilical cord blood shown to be important in fetal growth and development or associated with other toxic metals including the *H19* DMR regulating the *IGF2/H19* domain, the *MEG3* DMR regulating the *MEG3* domain, the *SGCE*/*PEG10* DMR positioned between *Epsilon Sarcoglycan and Paternally Expressed Gene 10*, the *NNAT, MEST and PEG.* We hypothesized Cd exposure in utero alters offspring DNA methylation levels in the DMRs regulating genomically imprinted *NNAT, MEST, PEG3, PLAGL1, PEG10, IGF2, H19*, and *MEG3* DMRs and that maternal Zn and Fe levels alter these associations.

## Methods

### Study participants

Study participants were pregnant women who were enrolled as part of the Newborn Epigenetic STudy (NEST), a prospective cohort study of women and their offspring with the overarching goal of investigating the effects of *in utero* exposures on epigenetic profiles and phenotypes in children. Between 2009 and 2011, pregnant women were recruited from five prenatal clinics and obstetric care facilities at Duke University and Durham Regional Hospitals. Details of participant accrual have been previously described [16, 17]. Eligibility criteria were age 18 years or older, pregnant and intending to use one of two participating obstetric facilities in Durham County for delivery. Excluded were women who planned to relinquish custody of the index child, move states in the subsequent 3 years, or had an established HIV infection. In the 18-month period between April, 2009 and 2011, 2,548 women were approached and 1,700 consented (66.7 % response rate). The present analyses are limited to the first 319 infant-mother pairs for whom maternal Zn, Fe and Cd blood levels and infant DNA methylation were measured. Maternal race, smoking status, BMI before pregnancy, parity, delivery route, and education were comparable in the 319 infant-mother pairs included in this study and the remainder of the cohort (p > 0.05). The study protocol was approved by the Institutional Review Boards of Duke and North Carolina State Universities.

### Data and specimen collection

Participants completed a self-administered questionnaire at the time of enrollment that included social and demographic characteristics, reproductive history, lifestyle factors, and anthropometric measurements. At study enrollment, maternal peripheral blood samples were collected; the mean gestational age at maternal blood draw was 12 weeks. Infant cord blood specimens were collected at birth. The leukocyte-containing buffy coat was isolated following centrifugation at 3500 × g for 20 min at 4 °C. Aliquots were prepared and stored at −80 °C.

### DNA methylation analysis

Genomic DNA from buffy coat from umbilical cord blood specimens was extracted using PureGene reagents (Qiagen; Valencia, CA) and treated with sodium bisulfite using the Zymo EZ DNA Methylation Kit (Zymo Research, Irvine, CA). Bisulfite treatment modifies the DNA by converting unmethylated cytosines to uracils but leaves methylated cytosines unchanged. Pyrosequencing was performed using a Qiagen Pyromark Q96 MD Pyrosequencer. Primers and PCR conditions have been previously described [18, 19]. The percent methylation for each CG dinucleotide was calculated using PyroQ CpG Software (Qiagen). The percent methylation was analyzed at multiple CpG sites for nine imprinted gene DMRs, including the maternally methylated *NNAT, MEST, PEG3, PLAGL1, PEG10* DMRs and the paternally methylated *IGF2, H19,* and *MEG3* DMRs.

### Measurement of Cd, Fe and Zn

Maternal Zn, Fe and Cd blood levels were measured in whole blood as nanograms per gram (ng/g; 1000 ng/g = 1025 ng/μl) using well-established solution-based ICP-MS methods [20–23]. Frozen maternal blood samples were equilibrated at room temperature, homogenized with a GlobalSpec laboratory slow shaker (GlobalSpec, East Greenbrush, NY) and ~0.2 mL aliquots were pipetted into a trace-metal-clean test tube and verified gravimetrically to ±0.001 mg using a calibrated mass balance. Samples were then spiked with internal standards consisting of known quantities (10 and 1 ng/g, respectively) of indium (In) and bismuth (Bi) (obtained from SCP Science), used to correct for instrumental drift. The solutions were then diluted using water purified to 18.2 MΩ/cm resistance (by a Milli-Q water purification system, Millipore, Bedford, Mass., USA) and acidified using ultra-pure 12.4 mol/L hydrochloric acid to result in a final concentration of 2 % hydrochloric acid (by volume). All standards, including aliquots of the certified NIST 955c, and procedural blanks were prepared by the same process.

Zn, Fe and Cd concentrations were measured using a Perkin Elmer DRC II (Dynamic Reaction Cell) axial field ICP-MS at the University of Massachusetts-Boston [20–23]. To clean sample lines and reduce memory effects, sample lines were sequentially washed with 18.2 MΩ cm resistance (by a Milli-Q water purification system, Millipore, Bedford, Mass., USA) water for 90 s and a 2 % nitric acid solution for 120 s between analyses. Procedural blanks were analyzed within each block of 10 samples, to monitor and correct for instrumental and procedural backgrounds. Calibration standards used to determine Zn and Cd in blood included aliquots of 18.2 MΩ cm resistance H_2_O, NIST 955c SRM, and NIST 955c SRM spiked with known quantities of each metal in a linear range from 0.025 to 10 ng/g. Standards were prepared from 1000 mg/L single element standards obtained from SCP Science, USA. Method detection limits were calculated according to the two-step approach using the t_99_S_LLMV_ method (USEPA, 1993) at 99 % CI (t = 3.71). The MDLs for Zn and Cd yielded values of 278 and 83 pg/g parts per trillion, respectively. Limits of detection (LOD) and limits of quantification (LOQ) according to Long and Winefordner (1983) were less than ~43 pg/g and 129 pg/g for Zn, respectively and less than ~17 pg/g and 51 pg/g for Cd, respectively.

### Measurement of folate

Maternal whole blood samples were sent to Craft Technologies (Wilson, NC, USA) for measurement of erythrocyte folate using a commercial kit, ID-Vit Folic acid (Immundiagnostic-ALPCO; Salem, NH, Ref KIF005) which uses the folate dependent strain *Lactobacillus rhamnosus* (taxon id 47715) in a 96 well format.

### Statistical analyses

Covariates considered for confounded were race/ethnicity (White, African American, Hispanic, and other), and parity (nulliparous and multiparous), physical activity (≥3 days per week, yes/no), and maternal smoking (yes, no, and quit during pregnancy). Maternal Zn, Fe and Cd blood levels were natural log transformed to achieve a normal distribution. Since at moderate levels, Zn and Fe have been shown to interact with Cd and to mitigate cellular, epigenetic and phenotypic effects in vivo and in vitro [9, 11, 24, 25], we explored these potential effects by categorizing Zn and Fe into high (top tertile) and low (bottom two tertiles) levels. We then modeled blood Cd on DMR methylation restricted to those with high and low levels of Fe or Zn. Factors evaluated for confounding were maternal age at delivery (less than 30 years, 30–39 years, and older than 40 years), the mother’s educational achievement (high school graduate/less and college/beyond), household income (less than $24,999, $25,000–$49,999, $50,000–$100,000, and more than $100,000 per year), and BMI (kg/m^2^, normal <25, overweight 25–30, obese 30–35, and extremely obese >35) calculated from self-reported maternal weight before pregnancy at last menstrual period and measured height at the study visit. Pre-pregnancy weight was verified with the hospital medical charts. The accuracy of retrospectively capturing weight was evaluated by abstracting weight from medical records of 237 cohort members who visited a Duke clinic within 6 months of their first prenatal visit for the pregnancy that made them eligible for the study, including a subset of 43 had visited within three months of their first prenatal clinic visit. The correlation between periconceptional self-reported and nurse-measured weight was 0.95 in the 237 and 0.98 in the 43 participants, (*p* < 0.0001). Parturition data examined included gestational age (preterm <258 days and term >259 days), delivery route (vaginal and C-Section) and birth weight (in grams). Given that maternal erythrocyte folate levels can affect DNA methylation, we used measurements of folate using methods previously described [15] based on a published protocol [26]. Since childhood obesity may vary by ethnicity/race, pre-pregnancy obesity, and sex, we also explored the potential for effect modification of the associations between Cd and DNA methylations and obesity in early childhood in African Americans, Hispanics and Whites. Final models were adjusted for maternal race, maternal smoking status, erythrocyte folate levels, parity, prenatal physical activity, infant sex and maternal pre-pregnancy BMI. All statistical analyses were conducted in Stata Version 13.0 (Stata Corp, 2013) using the xtmixed function, and findings were replicated in SAS. These analyses were repeated using logistic regression models where DNA methylation was dichotomized at the 4^th^ quartile, and weight at birth and age one year were categorized at <2500, 2500–4000 and >4000 g and <85^th^, 85- < 95^th^, and >95^th^ percentile, respectively, and findings were comparable.

## Results

### Cd and socio-demographic characteristics

Maternal Cd levels did not vary by maternal age, pre-pregnancy obesity, gestational age at delivery, or by sex and birth weight of offspring. However, Cd levels were somewhat higher among Hispanic and African American women compared to White women (*p* = 0.03) (Table 1). Cd blood concentrations were also higher among smoking mothers compared to nonsmoking mothers (*p* = 0.01), and were higher in women who delivered vaginally, compared to C-section deliveries (*p* = 0.02). These factors were accounted for in multivariable analysis.

**Table 1:** Distribution of maternal blood cadmium concentrations (ng/g) (*n* = 319)

Characteristic	Number	Mean	SD	*p*-value
Child Gender				0.73
Male	164	4.43	(6.66)	
Female	155	4.65	(5.53)	
Gestation Time				0.12
Preterm (<37 weeks)	39	3.07	(4.70)	
Normal(≥37 weeks)	280	4.74	(6.28)	
Birth Weight				0.61
Low Birth Weight (<2500)	31	4.67	(5.93)	
Normal Birth Weight(2500- < 4600)	281	4.58	(6.22)	
High Birth Weight (>4600)	5	1.84	(1.40)	
Maternal Age				0.35
Less than 30	187	4.93	(6.24)	
30-39	125	3.96	(6.08)	
40+	7	4.28	(2.94)	
Maternal Race				0.03
African American	111	4.71	(4.65)	
White	95	3.13	(5.07)	
Hispanic	101	5.44	(7.63)	
Other	12	6.48	(9.51)	
Maternal BMI				0.48
Less than 18.5	9	5.39	(7.46)	
18.5 to less than 25	111	4.20	(5.83)	
25 to less than 30	78	4.88	(5.90)	
30 to less than 35	39	2.95	(3.55)	
35+	29	3.87	(4.25)	
Maternal Smoking Status				0.01
Smoking Prior to Pregnancy	39	2.56	(3.13)	
Smoking During Pregnancy	46	6.44	(4.79)	
Never Smoke	225	4.42	(6.59)	
Delivery Route				0.02
Vaginal	198	5.16	(6.72)	
Cesarean section	120	3.50	(4.88)	

### Maternal Cd exposure and newborn DMR methylation

We evaluated the association between maternal Cd levels and DNA methylation at 9 differentially methylated regions of genomically imprinted genes in the offspring. We found a significant association between maternal Cd concentrations and altered methylation at the DMR regulating *PEG3* (β = 0.36, se = 0.17 *p* = 0.03). We also found associations of borderline significance between maternal Cd concentrations and altered methylation at the DMRs regulating *MEG3* (β = 0.44, se = 0.30, *p* = 0.14), and *NNAT* (β = 0.54, se = 0.32, *p* = 0.09) (Table 2).

**Table 2:** Regression coefficients, standard errors and *p*-values for the association between maternal blood cadmium exposure and offspring DNA methylation at DMRs imprinted genes

DMRs	All	Males	Females
	β, SE, *P*-value	β, SE, *P*-value	β, SE, *p*-value
*IGF2/H19*	−0.02, 0.18, 0.91	0.06, 0.25, 0.80	−0.20, 0.25, 0.42
*MEG3*	0.44, 0.30, 0.14	0.72, 0.41, 0.08	0.37, 0.42, 0.38
*MEST*	0.05, 0.22, 0.83	0.47, 0.29, 0.10	−0.34, 0.34, 0.31
*NNAT*	0.54, 0.32, 0.09	0.61, 0.45, 0.17	0.41, 0.48, 0.39
*PEG3*	0.36, 0.17, 0.03	0.09, 0.17, 0.61	0.55, 0.28, 0.05
*SGCE/PEG10*	0.01, 0.19, 0.98	−0.23, 0.26, 0.36	0.38, 0.28, 0.18
*PLAGL1*	−0.20, 0.34, 0.56	−0.42, 0.50, 0.40	−0.17, 0.48, 0.72

Associations may be specific to females at the *PEG3* (β = 0.55, *p* = 0.05) but not in males (β = 0.09, *p* = 0.61); whereas associations with the *MEG3* (β =0.72, se = 0.41, *p* = 0.08), and *MEST* (beta = 0.47, se = 0.29, *p* = 0.10) DMRs may be specific to males and less evident in females. No evidence for sex-specific association at other DMRs was found.

Despite higher levels of Cd in Hispanics, restricting these analyses by self-reported ethnicity/race and further adjusting for sex of offspring revealed other consistent associations between Cd exposure and regulatory DMR methylation (data not shown).

### Maternal Fe and Zn abundance in associations between Cd and DNA methylation at regulatory sequences of genomically imprinted genes

**Fig. 1:**
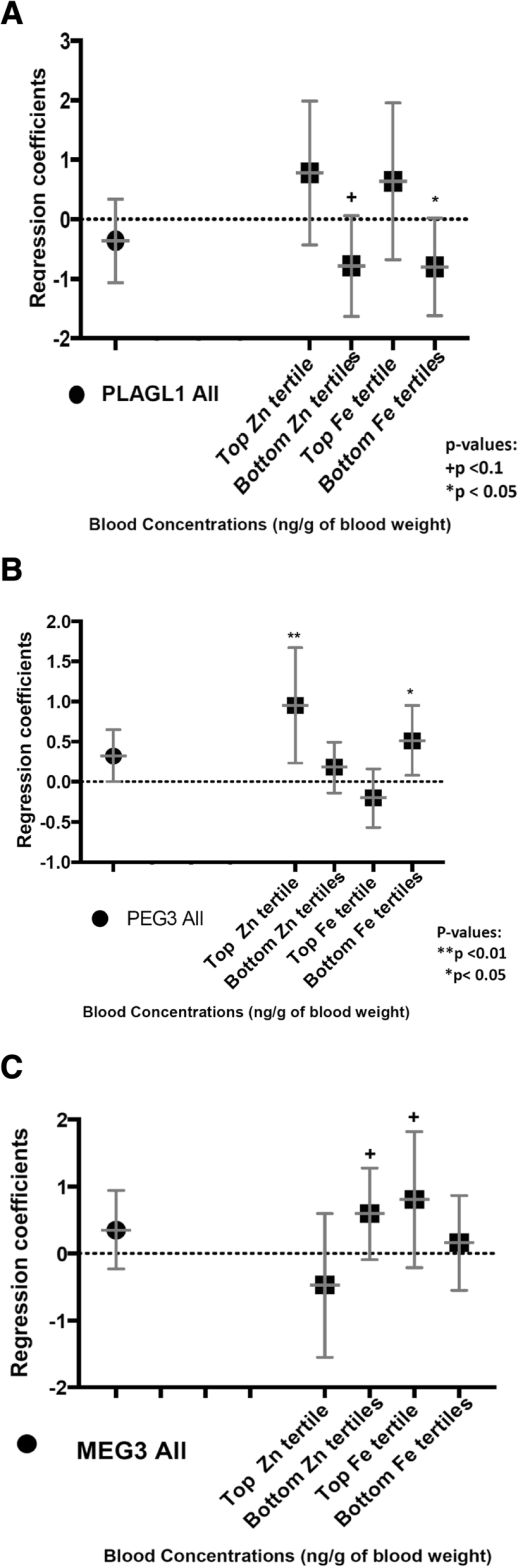
Regression coefficients for the associations between maternal Cd and DNA methylation at newborns imprinted genes. **a**. Infants born to women with high blood Cd concentrations but with lower Zn and Fe levels, had lower DNA methylation at the *PLAGL1* DMR. **b**. Maternal higher Zn and Cd concentrations were associated with higher methylation at the *PEG3* DMR in newborns. **c**. No associations were observed for *MEG3*

We also explored the extent to which maternal Zn and Fe altered the pattern of the associations observed between maternal Cd and offspring DNA methylation at the *MEG3, PLAGL1* and *PEG3*, by repeating the refined models, restricted to women and children with high concentrations of Zn or Fe (top tertile), and again among those with low levels (bottom two tertiles) (Fig. 1). We observed that higher Cd levels were associated with lower *PLAGL1* DMR methylation levels in women with lower Zn and Fe levels (Fig. 1a); the cross-product term for the interaction between Zn and Cd was *p* = 0.04, and that for Cd and Fe was (*p* = 0.37). We also found that higher *PEG3* DMR methylation levels were associated with higher Cd concentrations, but this association appeared limited to women with Zn levels in the top tertile (*p* < 0.05) (Fig. 1b). The cross-product term for the interaction between Cd and Zn was *p* = 0.01. This positive association between elevated Cd levels and higher *PEG3* methylation was similar in women with high and low Fe levels (Fig. 1b); the cross-product term for the interaction between Fe and Cd was *p* = 0.45. We also found no evidence that the association between Cd levels and *MEG3* DMR methylation varied by Fe and Zn nutrient abundance (Fig. 1c).

### Cd levels and birth weight

As Cd placental sequestration has been associated with fetal growth restriction; consequently, we also evaluated whether maternal Cd levels were associated with birth weight (Table 3). We found that higher maternal Cd concentrations in blood were associated with lower birth weight (β = −51.89, se = 24.20, *p* = 0.03).

**Table 3:** Associations between maternal cadmium levels and birth weight

	Adjusted associations of Log Cd and birth weight (*n* = 276)		
	β	se	p
*Maternal*			
Log Cd	−51.89	24.20	0.03
^a^Race/Ethnicity			
NH Black	−237.73	70.38	0.00
Hispanic	−164.34	72.27	0.02
NH Other	−222.72	134.09	0.10
Smoking			
Smoking during pregnancy	−173.52	106.27	0.10
Quit during pregnancy	−12.21	85.77	0.89
Pre-Pregnancy BMI	13.68	3.99	0.00
Prenatal physical Activity	5.23	64.31	0.94
Periconceptional antibiotic use	−94.61	60.38	0.12
*Offspring*			
Female	−107.72	52.19	0.04
Gestational Age at Delivery	26.48	2.19	0.00
Constant	−3996.97	633.10	0.00

Characteristics mutually adjusted

^a^Referents are non-Hispanic whites

## Discussion

While the known effects of Cd include replacement of essential protein cofactors, protein disruption/misfolding, generation of oxidative stress, and endocrine disruption, epigenetic mechanisms are emerging as a dynamic mechanistic framework for how the environment interacts with the genome to influence low birth weight and cardio metabolic risk in later life. However, regions of the human epigenome targeted by these ubiquitous toxic metals are still unknown. Moreover, there is limited empirical longitudinal human data on the role of essential metals such as Zn and Fe, previously shown to be antagonists to Cd absorption in animal models [24, 25]. We conducted an analysis to determine whether maternal Cd concentrations, are associated with birth weight, DNA methylation alterations at multiple DMRs regulating genomically imprinted genes after accounting for maternal race/ethnicity, antibiotic use, physical activity, parity, cigarette smoking and sex. We also evaluated the extent to which the abundance of the essential minerals, Zn and Fe modify this association.

Our key finding was that elevated Cd levels were significantly associated with higher DNA methylation at the DMR regulating *PEG3;* these associations varied by Zn or Fe circulating levels. We also found that higher maternal Cd levels were associated with lower birth weight. Our data support that early Cd exposure is gene-specific as DNA methylation of several other DMRs evaluated in newborns were not associated with prenatal levels of Cd. DNA methylation at these regulatory DMRs are established in gametogenesis and embryogenesis and may be vulnerable to availability/abundance of circulating essential metals, including Fe and Zn. DNA methylation marks here examined have previously been associated with fetal development. If these findings are replicated in a larger study and from a wider scope of regulatory regions, our findings suggest that nutritional manipulation during vulnerable periods in life could alter disease course, in Cd-exposed populations. Moreover, if these epigenetic marks are confirmed to be stable in humans, these regions could be developed as markers of periconceptional Cd exposure, for potential use in risk assessment.

Our data suggest that Cd concentrations during early pregnancy are associated with lower levels of newborn’s DNA methylation at the DMR regulating *PEG3,* and less consistently at *IGF2/H19* and *MEG3* and none with *PLAGL1* imprinted domains*. PEG3* is a paternally expressed imprinted gene, maps to chromosome 19q13.43 and encodes a zinc finger protein with a tumor suppressor function that plays a role in facilitating p53/c-*myc*-mediated apoptosis. *PEG3* also plays a critical role in brain development where it is mainly expressed in the mesencephalon and the pituitary gland [27]. Shifts in DNA methylation at the *PEG3* DMR have been shown to alter social and maternal nurturing behaviors in mice [27, 28] and to be associated with human cancer [19], presumably caused by a decrease in *PEG3* transcription [29, 30]. In support, a long term follow-up study, lower *PEG3* DMR methylation was recently associated with exposure to lead, a +2 toxic metal that tends to co-occur with Cd (Li et al., in press). Lower DNA methylation levels at the same *PEG3* DMR in males and *IGF2/H19* DMR in females have been associated with exposure to another environmentally abundant neurotoxin, lead, during the neonatal period (Li et al., in press).

While we know of no other study that has examined Cd exposure in relation to epigenetic dysregulation specifically targeting genomically imprinted genes, our findings that Cd exposure *in utero* is associated with DNA methylation differences is consistent with *in vitro* and *ex-vivo* systems studies showing that Cd is an effective inhibitor of DNA methyltransferase. Specifically, Cd initially induces DNA hypomethylation, although prolonged Cd exposure results in DNA hypermethylation and enhanced DNA methyltransferase activity [9]. In humans, gene-specific studies based on known gene function have shown DNA methylation alterations in response to Cd exposure [31, 32]; however, few have been conducted at birth to reflect the periconceptional environment [12]. Using the unbiased Illumina HumanMethylation450 BeadChip, maternal cigarette smoking, a common source of fetal Cd exposure, has been associated with altered DNA methylation at multiple CpG sites including 11 imprinted genes such as *AHRR*, *GFI1*, *IGF2* and *CYP1A1* in cord blood leukocyte DNA [33, 34]. In studies with small sample sizes [12, 35], *in utero* Cd exposure specifically was associated with CpG methylation differences in umbilical cord blood leukocyte DNA at multiple CpG sites mapping to genes involved in xenobiotic metabolism and inflammation [12], some of which may be sex-specific [36, 37]. Female-specific effects of Cd exposure have previously been reported in relation to birth weight, hypothesized to be due to lower iron in females that is associated with increased Cd intestinal absorption [38]. Therefore, while findings from these gene-specific and genome-scale studies do not include regions regulating *PLAGL1, MEG3* and *PEG3* examined here, together they support that *in utero* Cd exposure alters the epigenome.

Our findings that the association among maternal Cd and CpG methylation vary by Zn and Fe concentrations are consistent with the toxicity of these essential metals when present in excess, and reduced intestinal absorption reported in animal models. Both Zn and Fe have been shown to mitigate cellular, epigenetic and phenotypic effects *in vivo* and *in vitro* [9, 11, 23, 24, 39]. Mechanisms by which Fe or Zn may decrease harmful effects of Cd exposure remain unknown, although are an active topic of investigation. Studies suggest that marginal intakes of Zn, Fe, and Ca cause the accumulation of Cd in the duodenum, which results in a greater rate of Cd absorption and a greater accumulation in the internal organs [24, 25], which may influence fetal development and epigenetic dysregulation. However, these associations may also depend on other essential elements as well as age and sex. Cd may compete with essential metals for binding to transporter molecules. Indeed, Fe deficiency has been shown to upregulate Fe transporters and subsequently, increased Cd in duodenal mucosa [40, 41]. Deficiencies or excesses of these essential metals can contribute to epigenetic alterations and/or other mechanisms to influence phenotypes such as birth weight and childhood obesity [42], associations that may vary by race/ethnicity as our present results suggest. Zn and Fe are essential co-factor for many enzymes that epigenetically modify DNA and histones, and maternal Zn excess or deficiencies may program offspring growth trajectories by altering epigenetic modifications of DNA and histones [39, 43, 44] at labile loci. Together, our findings corroborate animal and *in vitro* data [9, 24, 25].

A strength of our study is its longitudinal design in early life, as few studies examine Cd exposure before birth. Because Cd and Zn measurements were made in erythrocytes at gestational age <12 weeks, and many heavy metals bind to erythrocytes (a 120 day lifespan), metal concentrations examined here likely represent those of the periconceptional environment. As nutrient concentrations vary by ethnicity (NHANES), another strength is the multiethnic composition of the cohort, enabling examination of the effects of Cd and Zn on the four major ethnic group in the US: Whites, Blacks, Hispanics and Asians. However, given race/ethnic heterogeneity, we cannot exclude the possibility that our inability to find statistically significant associations between altered methylation at other DMRs in response to Cd exposure could be due, in part, to our limited statistical power in race-specific analyses. While statistical power was adequate for overall analyses, we are underpowered for examining higher-order interaction by race. Also, we examined only eight DMRs of more than 65 genes currently known to be imprinted in humans, these 65 genes themselves represent only 1-5 % of the human genome [45]. Analysis of the methylation status of a wider spectrum of the epigenome will be required to clarify the spectrum of genes and pathways that contribute to the association between maternal Cd and DNA methylation alterations in newborns. Despite these limitations, our data suggest that maternal exposure to Cd is associated with aberrant DNA methylation at multiple regions previously associated with preterm birth [46], fetal growth restriction and neurodevelopmental disorders, as previously reported in other geographic regions [36, 47]. With replication, *PLAGL1* and *PEG3* could be considered among epigenetic regions perturbed by Cd exposure early *in utero*.

## Conclusions

Although epigenetic mechanisms have been proposed to link early Cd exposure to human health, human data are still limited, as epigenetic targets that may influence human health are still unknown. We present data consistent with the hypothesis that maternal Cd exposure in early pregnancy alters DNA methylation at multiple DMRs in offspring with sex and possibly race/ethnic-specific effects, and that Zn may mitigate these effects. We also contribute data suggesting that early Cd exposure is associated with lower birth weight. As our data were too limited for mediation analyses, larger studies are required to confirm these findings and to determine the role of other imprinted and non-imprinted genes in the associations between Cd, Fe and Zn exposure in epigenetic alterations with known methylation profiles, and low birth weight, a consistent risk factor for childhood obesity and other chronic diseases. Such efforts would contribute data to nutritional policy related to these ubiquitous toxic metals.

## Abbreviations

Cd: Cadmium
Fe: Iron
Zn: Zinc
DMR: Differentially methylated region
